# A Halotolerant Bacterium *Bacillus licheniformis* HSW-16 Augments Induced Systemic Tolerance to Salt Stress in Wheat Plant (*Triticum aestivum*)

**DOI:** 10.3389/fpls.2016.01890

**Published:** 2016-12-16

**Authors:** Rajnish P. Singh, Prabhat N. Jha

**Affiliations:** Department of Biological Science, Birla Institute of Technology and SciencePilani, India

**Keywords:** ACC deaminase, exopolysaccharide (EPS), *AcdS*, osmolytes, salt-stress, ERIC-PCR

## Abstract

Certain plant growth promoting bacteria can protect associated plants from harmful effects of salinity. We report the isolation and characterization of 1-aminocyclopropane-1-carboxylic acid (ACC) deaminase bacterium *Bacillus licheniformis* HSW-16 capable of ameliorating salt (NaCl) stress in wheat plants. The bacterium was isolated from the water of Sambhar salt lake, Rajasthan, India. The presence of ACC deaminase activity was confirmed by enzyme assay and analysis of *AcdS* gene, a structural gene for ACC deaminase. Inoculation of *B. licheniformis* HSW-16 protected wheat plants from growth inhibition caused by NaCl and increased plant growth (6-38%) in terms of root length, shoot length, fresh weight, and dry weight. Ionic analysis of plant samples showed that the bacterial inoculation decreased the accumulation of Na^+^ content (51%), and increased K^+^ (68%), and Ca^2+^ content (32%) in plants at different concentration of NaCl. It suggested that bacterial inoculation protected plants from the effect of NaCl by decreasing the level of Na^+^ in plants. Production of exopolysaccharide by the *B. licheniformis* HSW-16 can also protect from Na^+^ by binding this ion. Moreover, application of test isolate resulted in an increase in certain osmolytes such as total soluble sugar, total protein content, and a decrease in malondialdehyde content, illustrating their role in the protection of plants. The ability of *B. licheniformis* HSW-16 to colonize plant root surface was examined by staining the bacterium with acridine orange followed by fluorescence microscopy and polymerase chain reaction-based DNA finger printing analysis. These results suggested that *B. licheniformis* HSW-16 could be used as a bioinoculant to improve the productivity of plants growing under salt stress.

## Introduction

Soil salinity is one of the most important abiotic factors adversely affecting soil microbial activities and crop productivity. Reports suggest that >20% agricultural land worldwide is affected by salt (Pitman and Läuchli, 2002). It is estimated that the salinization will cause to the loss of 50% arability of agricultural land by middle of the 21st century (Wang et al., 2003). Saline soil adversely affects the plant growth and productivity by altering the normal metabolism of plants. In plants, one of the effects of salt stress is an increase in the pool of 1-aminocyclopropane-1-carboxylic acid (ACC), a precursor of ethylene, which results in accumulation of ethylene. Increase in the level of ethylene beyond a threshold level is termed as ‘stress ethylene’, which inhibits growth of root and shoot (Penrose and Glick, 2003), suppresses leaf expansion (Peterson et al., 1991), alters photosynthesis and photosynthetic components (Koryo, 2006), and promotes epinasty (Abeles et al., 1992). In addition to salt stress, other stressors such as flood, drought, wounding, pathogen attack, temperature stress, and mechanical stress also lead to significant rise in the level of endogenous ‘stress ethylene’ (Morgan and Drew, 1997; Stearns and Glick, 2003).

Association of plant growth promoting bacteria (PGPB) equipped with ACC deaminase activity can have a tremendous effect on mitigating plant growth inhibition resulting from stress ethylene. Under salt stress condition, much of the ACC exudes out from plant roots where ACC deaminase (ACCD) bacteria can sequester and degrade ACC to α-ketobutyrate and ammonia, thus, decreasing the building up of stress ethylene in plants. Improvement in the growth of groundnut and red pepper plants by ACCD containing *Pseudomonas fluorecens* TDK1 and *Bacillus* sp. under salt stress have been reported in earlier studies (Saravanakumar and Samiyappan, 2007; Siddikee et al., 2011). Similarly, Nadeem et al. (2007) also reported a protective effect of ACC deaminase containing bacteria *Pseudomonas syringae, Enterobacter aerogenes* and *Pseudomonas fluorescens* on the growth of maize under salt stress conditions. Another experimental report of Gamalero et al. (2008) also suggested a plant growth stimulatory effect of *Pseudomonas putida* UW4 and *Gigaspora rosea* BEG9 on the growth of cucumber under salt stress condition. PGPB isolated from saline habitats can be adapted to tolerate the salt and hence increase plant resistance to salt stress. Mayak et al. (2004) reported that salt tolerant ACC deaminase bacteria help plants to overcome stress effects. Hence, higher ACC deaminase activity could be one of the primary mechanisms by which bacteria support plant growth under salt stress (Saleem et al., 2007).

High salinity induces both ionic and osmotic stresses on plants by stimulating the generation of reactive oxygen species (ROS), which finally cause the deleterious oxidative damage (Munns and Tester, 2008; Gill and Tuteja, 2010) in plants. It also alters gene expression in plants at both transcription and translation level (Fujita et al., 2006). A number of genes reported to be up-regulated by salt stress in plants have also been shown to be up-regulated by other types of abiotic stressors. However, many elements of gene regulation remain to be poorly understood (Huang et al., 2012). The plants have developed several physiological and biochemical mechanisms to combat salt and other stress conditions. These mechanisms include osmotic adjustment by secretion of osmolytes, selective ion uptake and compartmentalization of ions (Shabala et al., 2014). Plants accumulate low molecular weight compatible solutes termed as ‘osmolytes’ such as proline, sugars, polyols, trehalose, and quaternary ammonium compounds (QACs) such as glycine betaine, alanine betaine, proline betaine, hydroxyproline betaine, choline *o*-sulfate, and pipecolate betaine for osmotic adjustment to cope with salt stress (Wang et al., 2003; Parida and Das, 2005; Ashraf and Foolad, 2007). Certain PGPB can enable plants to cope with the salt stress by augmenting above mentioned strategies which protect plants from the damages caused by salt stress. Osmolytes mediated protective mechanism is still not fully understood, however, it generally favors the osmotic adjustment (Colmer et al., 1995). In addition, its role in stabilizing membrane lipids (Hincha et al., 2003), maintenance of redox potential (Yancey, 2005), free radicals scavenging (Smirnoff and Cumbes, 1989), binding toxic metals (Sharma and Dietz, 2006), and induction of transcription factors under stress responses (Gupta et al., 2012) have also been reported. To cope with salinity-induced oxidative damage, an increase in osmolytes is essential for mitigation of oxidative stress (Liu et al., 2011).

Other than the accumulation of stress ethylene, an increase in salinity also causes restriction of water uptake and toxicity of Na^+^ (Mayak et al., 2004). Increased accumulation of Na^+^ in salt-rich agricultural land promotes senescence of older leaves (Munns and Tester, 2008). PGPB can overcome the harmful effects of salinity by maintaining a favorable ratio of Na^+^/K^+^ ions amenable for plants growth and development under high salt levels (Mayak et al., 2004). Moreover, exopolysaccharides secreted by PGPB also reduce the amount of Na^+^ available for plants uptake (Sutherland and Geddie, 1993; Lee and Han, 2005). It also acts as a protectant against ROS generated by stress conditions (Fujishige et al., 2006; Cooper, 2007) and is involved in other additional functions such as biofilm formation and colonization in plants (Cooper, 2007; Irie et al., 2012).

It is evident that PGPB can play an effective role in mitigating the unfavorable effect of stressors particularly salt stress through one or more mechanisms. Therefore, it requires the exploration of suitable bacterial strain(s) which can ameliorate salt stress in plants through one or more properties. The presence of plant growth promoting properties in PGPB includes nitrogen fixation, phosphate solubilization, phytohormone production, siderophore production, and biocontrol activities which help the plant grow in a sustainable manner. Therefore, the aim of the present work was to isolate and characterize salt tolerant bacterium with ACCD activity and other plant growth promoting properties, helpful in protecting plants from salt stress. In addition, evaluation of certain compatible solutes “osmolytes” responsible for the plant to tolerate salt stress in the presence of bacterial inoculation was also investigated.

## Materials and Methods

### Isolation of Bacteria

The hypersaline water was collected from different sites of Sambhar lake, India (26° 58′ N, and 75° 5′ E) using random sampling design. The sampling was done in the month of July (2013) and water samples were brought to the lab in ice packs. For the isolation of bacteria, water samples were serial diluted (decimally) up to 10^-9^ in sterile DF (Dworkin and Foster, 1958) minimal salt medium and plated on DF agar medium supplemented with 4% NaCl. Composition per liter of DF medium was as follows: KH_2_PO_4_ 4.0 g, Na_2_HPO_4_ 6.0 g, MgSO_4_⋅7H_2_O 0.2 g, glucose 2.0 g, gluconic acid 2.0 g, citric acid 2.0 g, trace elements: FeSO_4_⋅7H_2_O 1 mg, H_3_BO_3_ 10 μg, MnSO_4_⋅H_2_O 11.19 μg, ZnSO_4_⋅7H_2_O 124.6 μg, CuSO_4_⋅5H_2_O 78.22 μg, MoO_3_ 10 μg, pH 7.2). Bacterial colonies with distinct morphology were selected and further cultured on solid DF salt minimal medium containing 3 mM ACC (Sigma-Aldrich, USA) and incubated for 48 to 72 h at 30°C. Based on rich growth on selective medium, isolate HSW-16 was selected and subcultured several times on DF-ACC agar plate to ensure its ability to use ACC as nitrogen source. The test isolate HSW-16 was assayed for ACC deaminase activity and other plant growth promoting properties. Glycerol stock (15% w/v) of the isolate was prepared and stored at -70°C until further use.

### Biochemical Characterization

Biochemical tests such as Gram staining, starch agar test, IMViC (Indole, Methyl Red, Voges-Proskauer, Citrate Utilization test), and catalase were performed for the test organism following standard protocol (Prescott and Harley, 2002). Ability of the bacterium to utilize various carbon sources was tested using carbohydrate utilization test kit (KB 009, Himedia, India). The sensitivity to antibiotics gentamicin (30 μg), vancomycin (30 μg), kanamycin (5 μg), tetracycline (10 μg), and chloramphenicol (30 μg) was tested by placing antibiotic disks (HTM 002, Himedia, India) on bacterial culture spread on LB medium. Interpretation of result was done using zone inhibitive chart provided by the manufacturer.

### Amplification and Sequencing of 16S rRNA

To identify the bacterium at the molecular level, 16S rRNA gene was amplified by PCR using standard method (Singh et al., 2015). Amplified product was then purified using Qiaquick PCR purification kit (Qiagen, Germany). Sequencing of the resulting amplicons was done by Xcelris Genomics Labs Ltd (Xcelris Ahmedabad, India). The nucleotide sequence was analyzed by comparing with 16S rRNA genes available at GenBank database of National Centre for Biological Information (NCBI) using BLAST algorithm^1^ to find the closest match to type strain. The pairwise evolutionary distance between 16S rRNA gene sequence of the test isolate HSW-16 and related bacterial strains was calculated, and a phylogenetic tree was constructed by the Neighbor-Joining method using a software MEGA version 6.0 (Tamura et al., 2013). The bootstrap of 1000 replicates was used to cluster the associated taxa.

### Screening for Salt Tolerance

The tolerance of bacterium HSW-16 against salt stress was tested by growing it in tryptic soy broth (Himedia Laboratories, India) amended with different concentrations of NaCl (2-11% w/v). After 24 h, absorbance of the culture was determined at 600 nm in a spectrophotometer (Jasco Corporation, Japan). Bacterial culture was inoculated in triplicate. The uninoculated medium was used as blank.

### ACC Deaminase Assay

The test organism was grown in 15 ml of Tryptic soy broth (Himedia, India) to late log phase at 200 rpm for 24 h at 30°C. Then the cells were harvested by centrifugation, washed with 0.1 M Tris-HCl (pH 7.6) and incubated overnight in 7.5 ml DF-minimal medium containing 3 mM ACC as sole nitrogen source. The bacterial cells were returned to shaking water bath for induction of ACCD at 200 rpm for 24 h at 30°C. Then, cells were harvested by centrifugation, washed with 0.1 M Tris-HCl (pH 7.6), and resuspended in 600 μl of 0.1 M Tris-HCl (pH 8.5). Thirty microliter of toluene was added to the cell suspension and homogenized for 30 s. At this point, 100 μl of toluenized cells were set aside for protein assay and stored at 4°C. The remaining toluenized cell suspension was used immediately for ACC deaminase assay. ACC deaminase activity was assayed by measuring the amount of α-ketobutyric acid produced by the hydrolytic cleavage of ACC following the protocol of Penrose and Glick (2003). The quantity of α-ketobutyrate (KB) was determined at 540 nm by comparing an absorbance of the test sample with a standard curve of pure α-ketobutyrate (Sigma-Aldrich, USA).

### Amplification and Sequencing of *AcdS* Gene

Total genomic DNA of HSW-16 was isolated by Qiagen genomic DNA isolation kit (Qiagen, USA). *AcdS*, the structural gene encoding ACCD was amplified by polymerase chain reaction (PCR) using universal primers (Duan et al., 2009). Sequences of primers were: (F) 5′-GGCAAGGTCGACATCTATC-3′ and (R) 5′-GGCTTGCCATTCAGCTATG-3′. Amplification was performed in a final volume of 50 μl containing genomic DNA (50 ng), 20 picomoles each of forward and reverse primers, 200 μM of each dNTP (Genei, India), 1X Taq polymerase buffer and 2.5 U of Taq DNA polymerase (Genei, India). The thermal profile of PCR was set with an initial denaturation at 94°C for 3 min, 35 cycles of denaturation at 94°C for 1 min, annealing at 58°C for 1 min and primer extension at 72°C for 3 min, followed by a final extension at 72°C for 5 min in a thermal cycler (T100, BioRad, USA). The *AcdS* gene sequence was determined by sequencing of PCR product at Xcelris Genomics Labs Ltd (Xcelris, India). The gene sequence was analyzed using BLASTn search program^2^ for nucleotide sequence homology. The *AcdS* nucleotide sequence of *Bacillus* sp. and other bacterial strains was obtained from the NCBI database. The nucleotide sequences were aligned by ClustalW using MEGA 6.0 software and a Neighbor-Joining (NJ) tree with the bootstrap value 1000 was generated using the software.

### Screening for Plant Growth Promoting Traits

#### Production of Phytohormones

The test isolate HSW-16 was tested for production of indole-3-acetic acid (IAA) following the method of Gordon and Weber (1951). Briefly, the culture was grown in Nutrient broth containing 100 μg ml^-1^ tryptophan at 30°C with shaking at 180 rpm in a bacteriological incubator. After growth for 72 h, the culture was harvested by centrifugation at 10,000 *g* for one min. One ml of resulting supernatant was mixed with 2 ml Salkowsky’s reagent (35% HClO_4_, 0.01 M FeCl_3_) and kept at room temperature in the dark for 20 min. Optical density (OD) was measured spectrophotometrically in a UV-visible spectrophotometer at 530 nm (Jasco, Japan). The concentration of IAA was determined from the standard curve of pure IAA (Merck, Germany). It was also tested for another phytohormone gibberellic acid using the method of Holbrook et al. (1961). Briefly, HSW-16 was grown in nutrient medium, centrifuged, and pH of the culture supernatant was adjusted to 2.5 using 2 N HCl and extracted with equal volume of ethyl acetate for 2 to 3 times in a separating funnel. After mixing 1.5 ml extract with 0.2 ml of 5 mM potassium ferrocyanide and 9.8 M HCl, absorbance was measured at 254 nm in a UV-Visible spectrophotometer (Jasco, Japan) using gibberellic acid (10-100 μg ml^-1^) as standard.

#### Mineral Phosphate Solubilizing Activity

Phosphate solubilizing activity was screened by growing the test isolate on National Botanical Research Institute’s Phosphate Medium (NBRIP) containing insoluble tricalcium phosphate [composition per liter: glucose 10 g, Ca_3_(PO_4_)_2_ 5 g, MgCl_2_.6H_2_O 5 g, MgSO_4_.7H_2_O 0.25 g, KCl 0.2 g, (NH_4_)_2_SO_4_ 0.1 g, pH 7.0] (Mehta and Nautiyal, 2001). The culture was spot inoculated on NBRIP-agar plate containing bromophenol blue and incubated at 30°C for a week. Observation of halo zone around bacterial growth on the plate was considered as positive for phosphate solubilization activity (Frey-Klett et al., 2005). Solubilized phosphate was quantified according to the method of Ames (1964) keeping the various concentration of K_2_HPO_4_ as standard.

#### Siderophore Production

For the test of siderophore production, 2 μl overnight-grown culture of HSW-16 was spot inoculated on chrome azurole S (CAS) agar plates (Schwyn and Neilands, 1987) and incubated at 30°C for 4-5 days. After the experimental period, bacterial colony was observed for the appearance of orange color around its growth. The experiment was performed in triplicate.

#### Nitrogen fixation

In order to test the ability of HSW-16 to fix nitrogen, a preliminary test for nitrogen fixation was done by growing it in minimal medium devoid of fixed nitrogen sources. HSW-16 was grown in LB media overnight and harvested at 8,000 *g* for 5 min. Bacterial pellet was washed two times with phosphate buffer saline (PBS). The JNFb^-^ agar plates (Döbereiner, 1995) were streaked with above culture and incubated at 28°C for 4 days and appearance of bacterium growth was observed as qualitative evidence of atmospheric nitrogen fixation. Composition of JNFb^-^ per liter was: malic acid 5.0 g, K_2_HPO_4_ 0.6 g, KH_2_PO_4_ 1.8 g, MgSO_4_⋅7H_2_O 0.2 g, NaCl 0.1 g, CaCl_2_ 0.02 g, 0.5% of bromothymol blue in 0.2 N KOH, vitamin solution 2 ml, micronutrient solution 1 ml, 1.64% Fe⋅EDTA (w/v) solution 2 ml, and KOH 4.5 g. One-hundred milliliters of vitamin solution contained 10 mg of biotin and 20 mg of pyridoxal-HCl. The micronutrient solution contained (per liter) CuSO_4_ 0.4 g, ZnSO_4_⋅7H_2_O 0.12 g, H_3_BO_3_ 1.4 g, Na_2_MoO_4_.2H_2_O 1.0 g, and MnSO_4_.H_2_O 1.5 g, pH was adjusted to 5.8. Sub-culturing was repeated three times to ensure diazotrophy in given isolate. In addition, *nif H* gene was amplified using specific primers: Pol F (5′-TGCGAYCCSAARGCBGACTC-3′) and Pol R (5′-ATSGCCATCATYTCRCCGGA-3′) (Sigma–Aldrich), where Y = C/T, S = G/C, R = A/G, B = G/T/C (Poly et al., 2001). PCR reaction mix contained 1× Taq DNA polymerase buffer, 50 pmol of each primer, 125 μM each dNTP, 1 U Taq DNA polymerase and 50 ng of template DNA. Thermal cycling used were: 94°C for 5 min; 30 cycles of 94°C for 1 min, 55°C for 1 min, and 72°C for 30 s followed by extension at 72°C for 5 min. The amplified product was analyzed on 1.8% agarose gel (w/v).

#### Ammonia Production

The freshly grown culture of the test organism was inoculated into 10 ml peptone water and incubated for 48 h at 37°C. After the bacterial growth, Nessler’s reagent (0.5 ml) was added to each tube. Development of brown to yellow color was observed as a positive for ammonia production (Cappuccino and Sherman, 1992). The uninoculated medium was used as blank for comparison.

#### Antagonism Test

Antagonistic activity of HSW-16 was evaluated by using agar well diffusion method against pathogenic fungal species namely *Aspergillus flavus, Candida albicans, Colletotrichum capsici, Fusarium oxysporum, Fusarium moniliforme, Fusarium graminearum*, and *Penicillium citrinum.* Antagonistic activity against certain bacterial pathogens such as *Bacillus cereus, Erwinia carotovora, Enterobacter* sp., *Escherichia coli, Klebsiella pneumoniae*, and *Staphylococcus aureus* was also determined. Standard bacterial/fungal strains were procured from Microbial type culture collection (MTCC), Chandigarh, India. Briefly, freshly grown cultures of fungal and bacterial species were spread on potato dextrose agar and tryptic soy agar plates, respectively. After adsorption, well size of 6 mm was made by metallic borer and filled with 100 μl (10^8^ CFU/ml) of the freshly grown culture of HSW-16 in tryptic soy broth medium. The plates were incubated for 7 days at 28°C for fungal species and 24 h at 37°C for bacteria. Antagonistic activity was determined by measuring zone of inhibition for which parameter used as: <10 mm = poor (+), between 10 to 20mm = good (++).

### Exopolysaccharide (EPS) Production Assay

For EPS assay, HSW-16 was inoculated into an Erlenmeyer flask containing 200 ml broth supplemented with 4% NaCl and grown for 5 days at 25°C with shaking at 150 rpm. Growth medium consisted following constituents (per liter): yeast extract 10 g, casamino acids 7.5 g, trisodium citrate 3.0 g, KCl 2.0 g, MgSO_4_.7H_2_O 20 g, MnCl_2_.4H_2_O 0.36 mg, and FeSO_4_.7H_2_O 50 mg (Nicolaus et al., 1999). After incubation, the culture was heated at 100°C for 15 min to denature EPS-degrading enzyme and then centrifuged at 9,000*g* for 30 min at 4°C. Three volume of chilled isopropanol was added to 1 vol of the supernatant and kept at 4°C for overnight precipitation. The precipitate was washed with 70% ethanol (v/v), and kept for drying in a desiccator. Residual protein was removed from the sample using 20% (v/v) trichloroacetic acid (TCA) and precipitate was redissolved in ultra-pure (Milli-q) water. The resultant sample was dialyzed to remove extra salts. Purified EPS was quantified using phenol-sulphuric acid test (Dubois et al., 1956). Detection of functional groups of purified EPS was done by FTIR (Fourier transform infrared spectroscopy) (Brucker Tensor -27) following micro-potassium bromide (KBr) pellet method. The pellet for infrared analysis was obtained by grinding a mixture of 2 mg exopolysaccharide with 200 mg dry KBr followed by pressing the mixture into a 16 mm diameter mold. The FTIR spectra were recorded in the region of 4000-400 cm^-1^.

#### Motility Test

Motility of bacteria is involved in the colonization behavior. Therefore, test organism was tested for its motility following the standard protocol of Connelly et al. (2004).

##### (i) Swimming

Tryptone swim plates containing 1% tryptone (w/v), 0.5% NaCl (w/v), 0.3% agar (w/v) were spot inoculated with test organism using a sterile toothpick and incubated for 16 h at 25°C. Motility was then assessed qualitatively by examining the circular turbid zone formed by the bacterial cells migrating away from the point of inoculation.

##### (ii) Swarming

Cells were point inoculated with a sterile toothpick on swarm plates and incubated at 30 °C for 24 h. Medium used for the test of swarming consisted of 0.5% Bacto-agar (w/v) and 8 g l^-1^ of nutrient broth (both from Difco, USA) supplemented with 5 g l^-1^ of dextrose.

##### (iii) Twitching

Cells were stab inoculated with a toothpick through a thin (approximately 3 mm) LB agar layer containing 1% agar (w/v) to the bottom of the Petri dish. After incubation for 24 to 48 h at 30°C, a hazy zone of growth at the interface between the agar and the polystyrene surface was observed

### Effect of HSW-16 on Plant Growth Under NaCl Stress Condition

#### Physiochemical Characteristics of Soil

Soil sample used for plant growth studies was analyzed for its various physico-chemical properties. The pH and electrical conductivity of soil were analyzed by digital pH and EC meter on a 1:2.5 ratio of soil and water, respectively. Estimation of organic carbon was done by the method of Walkey and Black (1934) using 1N potassium dichromate for titration and 0.5 N ferrous ammonium sulfate for back titration. Available soil phosphorus (Olsen P) was determined by chlorostannus-reduced molybdophosphoric blue color method after extraction with 0.5 M sodium bicarbonate as described by Olsen et al. (1954). Available nitrogen, potassium, and other micronutrients (Fe, Cu, Zn, and Mn) were estimated by the method of Jackson (1967).

#### Preparation of Bacterial Inoculum and Seed Treatment

The halotolerant bacterium HSW-16 was tested for its plant growth promoting effect on wheat (*Triticum aestivum* L., variety C-309) plants. Preparation of bacterial inoculum and seed bacterization was performed according to Penrose and Glick (2003) with slight modification. Briefly, HSW-16 was grown in 15 ml DF medium with 3 mM ACC as sole nitrogen source with 4% NaCl (w/v) for optimum growth of the isolate. The culture was incubated for 24 h at 30°C with shaking at 130 rpm in a bacteriological incubator (Labtech, India). After incubation, cells were harvested and re-suspended in 0.5 ml sterile 0.03 M MgSO_4_ and diluted two to three times to obtain the bacterial population of 1 × 10^8^ CFU/ml. Wheat seeds were surface sterilized by treating with 70% ethanol (v/v) for 2 min followed by three times washing with sterilized water. Then, the seeds were subjected to 1% (w/v) sodium hypochlorite (NaOCl) solution for 3 min followed by three consecutive washing with sterile water to remove all traces of sodium hypochlorite. Twenty seeds were treated with above-mentioned bacterial suspension for 1 h. Seeds treated with sterile 0.03 M MgSO_4_ served as a control. Following treatment, twenty seeds were sown in plastic pots filled with soil in a plant growth chamber (Labtech, India) with 16:8 day/night photoperiod up to 15 days at 28 ± 2°C with a humidity of 65-70%. The soil used for the pot studies was sterilized by autoclaving at 121°C for 1 h for three consecutive days and 300 g sterilized soil was filled in each plastic pot. Hoagland medium supplemented with NaCl (150 mM, 175 mM, 200 mM) was used for providing nutrient as well as imposing the salt stressorss (Hoagland and Boyer, 1936). Pots were arranged in completely randomized block design with three replications in each treatment. The experiment was conducted for 15 days after the germination of seeds. The growth of plants was measured in terms of root length, shoot length, fresh weight, and dry weight. For measurement of Chlorophyll a/b, fresh leaf samples (1 g) were homogenized in 80% acetone (v/v), and pigments were extracted and quantified (Duxbury and Yentsch, 1956). The absorbance at 480, 510, and 663 nm was measured on a UV–Vis spectrophotometer (Jasco, Japan). Five randomly selected plants from each experimental set up were also used for tests namely ionic accumulation, analysis of osmolytes, and confirmation of colonization as described in following sections.

#### Ionic Accumulation Analysis

For analysis of ionic elements, 1 g of shoot tissue from each sample was ground in liquid N_2_ and digested in 100 ml mixture of perchloric acid, sulphuric acid, and distilled water with the ratio of 10:1:2, respectively. Twenty milliliter of digested sample was used for analysis of Na^+^, K^+^, and Ca^2+^ by an Atomic Absorption Spectrophotometer (AAS 2380, Perkin Elmer, USA) at National Horticultural Research and Development Foundation (Nashik, India). For accuracy and precision, each sample was analyzed in triplicate sets.

#### Biochemical Analysis for Osmolytes in Plant After NaCl and Bacterial Inoculation

Proline content in the leaves was determined following the standard protocol (Bates et al., 1973) with minor modifications. A 0.5 g fresh leaves of experimental plant samples were homogenized in 3 ml of 5% (w/v) sulfosalicylic acid and centrifuged at 8,500*g* for 10 min. To 500 μl of the resulting supernatant, 1 volume of water and 2 volumes 2% ninhydrin (w/v) were added. The mixture was incubated at 100°C for 30 min. After cooling, an equal volume (2 ml) of toluene was added to the mixture and upper aqueous phase was used for measuring absorbance at 520 nm in a spectrophotometer (Jasco, Japan). The proline content was calculated by comparing with a standard curve of pure L-proline (Sigma–Aldrich, USA).

Total soluble sugar (TSS) was estimated by anthrone reagent according to Irigoyen et al. (1992). 0.1 ml of alcoholic leaf extract prepared by homogenized 0.5 g leaf with 3 ml of 80% ethanol (v/v) was mixed with 3 ml of freshly prepared anthrone reagent and placed in a boiling water bath for 10 min. The absorbance of the resultant sample was measured at 620 nm. 20-400 μg ml^-1^ of glucose was used as a standard for making calibration curve for quantification of soluble sugar present in plants. Lipid peroxidation was determined by estimating the malondialdehyde (MDA) content produced by the thiobarbituric acid (TBA) reaction as per Hodges et al. (1999) with minor modification. Briefly, 1 ml of alcoholic extract (80%) prepared with 0.5 g of leaves was mixed with 1 ml of 0.5% (w/v) TBA containing 20% (w/v) TCA. The mixture was heated up to 90°C for 30 min in a water bath. After cooling at the room-temperature sample was centrifuged at 4,500 *g* for 5 min and absorbance was measured at 400, 532, and 600 nm. After subtracting the non-specific absorbance, the MDA concentration was determined by its molar extinction coefficient (155 nm ^-1^cm^-1^) and the results were expressed as mmol g^-1^ FW.

Similarly, alcoholic extract of shoot tissue was used for measuring the auxin content as per Andreae and Ysselstein (1959). One ml of alcoholic extract of shoot tissue was mixed with 2 ml of Salkowsky reagent in the dark and incubated for 20 min. The absorbance was measured at 535 nm and compared with the standard curve of IAA. For the estimation of total protein content (TPC), protein was extracted by homogenizing 0.5 g plant tissue in extraction buffer containing Tris-HCl 50 mM (pH 8.3), EDTA 1 mM, DTT 3 mM, ascorbic acid 0.08% (w/v), and PMSF 1 mM and quantified by Bradford method (Bradford, 1976).

#### Test of Colonization

For monitoring the colonization of bacterium, soil particles adhering to the root surface were gently removed. The roots were cut into 1 cm long segments and 1 g of root segments were dipped into 5 ml of sterilized PBS buffer, and vortexed 5-6 times to release the bacteria into the buffer. Dilutions of bacterial suspensions were poured on Nutrient agar to evaluate the population of the indigenous bacterium. The colony forming units were counted after 24 to 48 h of incubation at 28 ± 1°C. For confirming identity of colonized bacterium, enterobacterial repetitive intergenic consensus- (ERIC) PCR was performed in a 50μl reaction volume containing 50 ng bacterial DNA, 125 μM each dNTP (Genei, India), 1 X Taq DNA polymerase buffer (Genei, India) with 1.5 mM MgCl_2_, 20 pmol of each primers and 1.5 U of Taq DNA polymerase in a DNA thermal cycler (T 100, BIO-RAD, USA). Primers of ERIC 1R F′ (ATGTAAGCTCCTGGGGATTCAC) and ERIC 2R R′ (AAGTAAGTGACTGGGGTGAGCG) (Sigma-Aldrich, USA) were used for amplification. The thermal cycling condition was: initial denaturation for 3 min at 94°C, 35 cycles each consisting of denaturation for 1 min at 94°C, primer annealing for 1 min at 52°C and extension at 72°C for 5 min and a final extension of 7 min at 72°C. PCR product was analyzed on 2% agarose gel containing 0.5 μg ml^-1^ ethidium bromide using a gel documentation system (Bio-Rad, USA).

### Statistical Analysis

Data on the growth parameters of wheat was analyzed by a DPS statistical software package (version 11.0) by one-way ANOVA followed by Student’s *t*-test (*p* < 0.05). The statistical analysis was performed between 2 × 4 groups, by taking one groups of control and other with bacterial inoculated. Statistical difference between control and HSW-16 mean at each treatment were assessed by Student’s *t*-test (*p* < 0.05).

## Results

### Isolation, Biochemical Characterization, and Identification of HSW-16

**FIGURE 1:**
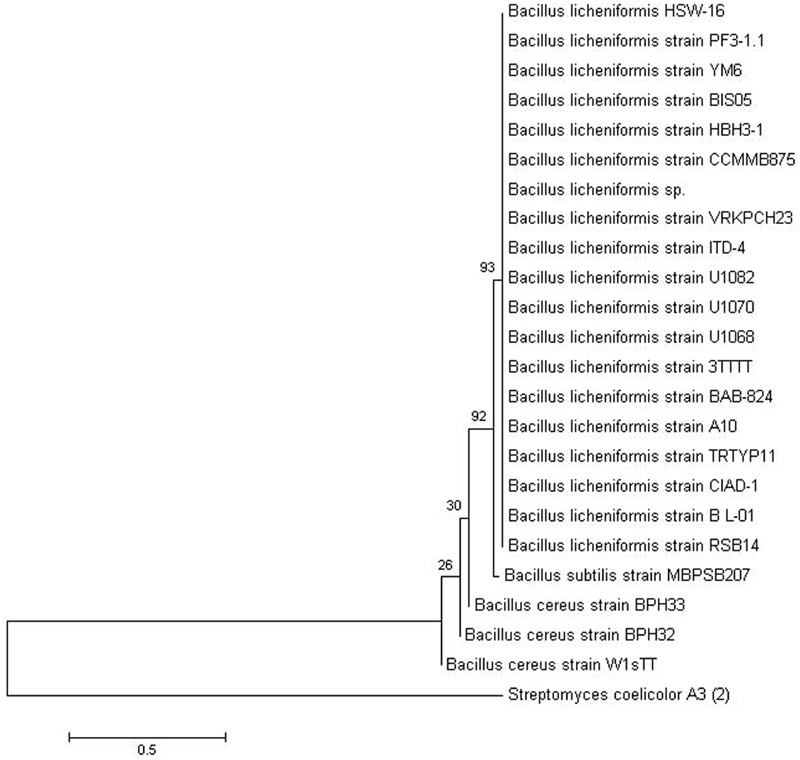
**Phylogenetic tree showing the relationship of *Bacillus licheniformis* HSW-16 to closely related bacteria.** The 16S rRNA gene sequence of closely related species was obtained from NCBI GenBank database. 16S rRNA gene of *Streptomyces coelicolor* A3 was taken as an outgroup. The tree was obtained using neighbor joining method of software package Mega version 6.0 at the bootstrap value of (*n* = 500).

Based on colony morphology, overall 13 different bacterial isolates were recovered from hypersaline Sambhar lake water. Out of these 13 isolates, one isolate namely HSW-16 showing consistent growth on minimal medium supplemented with ACC as the sole source of nitrogen was selected for detailed characterization. The ACC deaminase activity of HSW-16 was determined as 267.50 ± 19 nmol of α-KB mg^-1^ protein h^-1^. Basic microbiological and biochemical tests of the isolate HSW-16 indicated that it is a Gram-positive bacterium, which showed positive results for the test of Voges-Proskauer, lipase, nitrate reductase and negative for indole, methyl red, amylase, catalase, and urease. Moreover, antibiotic sensitivity profiling of HSW-16 showed its resistance to chloramphenicol, kanamycin, vancomycin and sensitivity to tetracycline and gentamicin (**Table 1**). The test organism HSW-16 was able to utilize various carbon sources such as xylose, maltose, fructose, dextrose, galactose, raffinose, trehalose, sucrose, L-arabinose, mannose, glycerol, salicin, inositol, sorbitol, mannitol, adonitol, α-Methyl-D-glucoside, rhamnose, cellobiose, ONPG, esculin hydrolysis, D-arabinose, sorbose, and inulin (**Table 1**). The carbohydrate utilization pattern of the test organism tallied with a standard strain of *B. licheniformis* reported by Salkinoja-Salonen et al. (1999). The tolerance of HSW-16 to NaCl was also assessed which showed NaCl tolerance in the range of 2-11% while optimal growth was observed at 8% of NaCl. To ascertain taxonomic affiliation of HSW-16 on a molecular level, it was subjected to 16S rRNA gene sequence analysis, which showed its closest match of 99% to the 16S rRNA gene sequence of *B. licheniformis* strain PF3-1.1 (**Figure 1**). It indicates that the test isolate probably belongs to *B. licheniformis.* The obtained sequence was submitted to the NCBI Genbank under the accession number KJ950717.

**Table 1:** Biochemical and physiological characteristic feature of isolate HSW-16.

Characteristic (s)	Activity	Carbohydrate	Utilization
Gram test	+	Sucrose	+
Indole	-	L-arabinose	+
MR	-	Sodium gluconate	-
VP	+	Glycerol	+
Amylase	-	Salicin	+
Lipase	+	Dulcitol	-
Urease	-	Inositol	+
Catalase	-	Sorbitol	+
Nitrate reductase	+	Mannitol	+
+ positive, - negative		Adonitol	+
Antibiotic resistance		Arabitol	-
Chloramphenicol	+	Erythritol	-
Tetracyclin	-	a-methyl-D-glucoside	+
Kanamycin	+	Rhamnose	+
Gentamycin	-	Cellobiose	+
Vancomycin	+	Melezitose	-
- sensitive, + resistant		a-methyl-D-mannoside	-
Carbohydrate	Utilization	Xylitol	-
Lactose	-	ONPG	+
Xylose	+	Esculin hydrolysis	+
Maltose	+	D-Arabinose	+
Fructose	+	Citrate utilization	-
Dextrose	+	Malonate utilization	-
Galactose	+	Sorbose	+
Raffinose	+	Inulin	+
Trehalose	+	Mannose	+
Melibiose	-		

### Molecular Characterization of ACC Deaminase

**FIGURE 2:**
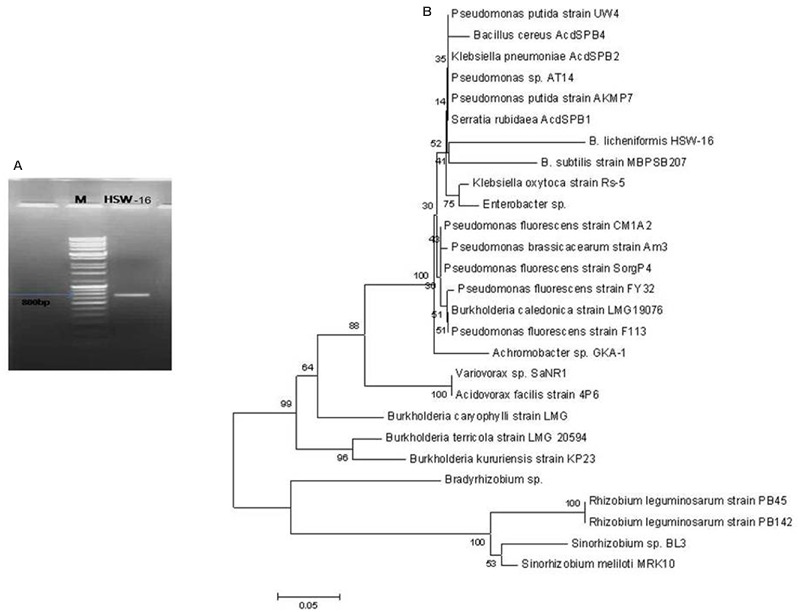
**Amplification of *AcdS* gene of *B. licheniformis* HSW-16. (A)** Shows amplicon of *AcdS* (800 bp) of the isolate in Lane HSW-16 and DNA ladder mix (Fermentas, SM0331) in Lane M. **(B)** represents dendrogram based on *AcdS* gene sequence of test isolate and other species. Neighbor-joining method was performed using the software packages Mega version 6.0 at the bootstrap value of *n* = 1000.

An 800 bp amplicon was obtained by PCR from genomic DNA of the isolate HSW-16 using *AcdS* gene specific primers (**Figure 2A**). Sequence analysis of the amplicon confirmed its identity where it showed 98% similarity with *AcdS* sequence of *B. cereus* AcdSPB4. The obtained sequence was submitted under the accession number KM501059. Sequence analysis of *AcdS* gene obtained from HSW-16 and other bacterial species revealed that *AcdS* sequence of HSW-16 is closely related to *B. cereus* AcdSPB4, *B. subtillis*, and strains belonging to other genera including *Klebsiella, Pseudomonas, Burkholderia, Achromobacter*, and *Serratia.* It also showed 99% similarity with one of the most characterized ACCD bacteria *Pseudomonas putida* UW4 (**Figure 2B**). Nucleotide sequence analysis suggests that *AcdS* gene found in *B. licheniformis* HSW-16 encodes a true ACC deaminase. Phylogenetic analysis revealed that nucleotide sequence of *AcdS* of genus *Bacillus* clustered together along with other species belonging to genera *Serratia, Klebsiella*, and *Pseudomonas*. The accuracy of placement in the tree of HSW-16 and MBPSP207 is possibly mitigated due to the use of partial sequences in the alignment.

### Plant Growth Promoting Features

**FIGURE 3:**
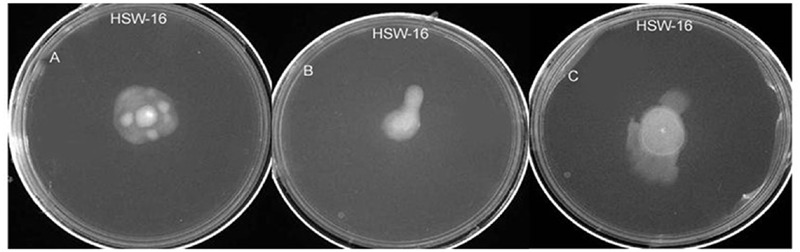
**Test of various types of motility in *B. licheniformis* HSW-16 using agar-based method. (A–C)** Represent swimming, swarming, and twitching, respectively.

The test organism *B. licheniformis* HSW-16 formed a clear zone on solid agar medium supplemented with an insoluble form of phosphate (tricalcium phosphate) which indicated mineral phosphate solubilizing activity. Phosphate solubilization was quantified after 72 h of growth which showed solubilization of 11.04 ± 2.44 μg ml^-1^ phosphate. For phytohormone production, it showed positive for the production of IAA while negative for gibberellic acid. It produced 2.89 ± 0.03 μg ml^-1^ IAA after 72 h of bacterial growth. Ability to fix atmospheric nitrogen was tested by growing it on selective medium lacking any source of fixed nitrogen (**Table 2**). Continuous growth for several generations on N^-^ medium indicated its ability to fix atmospheric nitrogen fixation. Furthermore, the amplification of the *nifH* gene supports the nitrogen-fixing potential of HSW-16 at the molecular level. The desired band of 350 bp corresponding to the *nifH* gene was obtained by using universal primers for the *nifH* gene (**Supplementary Figure S1**). In addition, it was also positive for the test of ammonia production (**Table 2**). However, it gave a negative result for the test of siderophore production. Biocontrol potential of test organism was evaluated by testing antagonistic activity against various fungal and bacterial pathogens. The test organism HSW-16 inhibited the growth of *Aspergillus flavus, Fusarium oxysporum, Fusarium graminearum*, and *Penicillium citrinum.* Among the bacterial pathogens tested, it was found inhibitory against *Enterobacter* sp., *Klebsiella pneumoniae, Erwinia carotovora*, and *Escherichia coli* (**Table 3**). The test isolate showed the swimming, swarming and twitching motility (**Figure 3**)

**Table 2:** Plant growth promoting traits of HSW-16.

Plant growth promoting traits	Activity
ACCD activity (nmol of α-KB mg^-1^ Pr.h^-1^)^∗^	267.50 ± 19
1AA production (μg ml)	2.89 ± 0.03
*t* -1n Phosphate solubilization (μg ml)	11.04 ± 2.44
Gibberelic acid production	-
Growth on N-free medium	+
Siderophore index	-
Ammonia production	+

*^∗^Enzyme activity was assessed after 72 h of bacterial growth.*

**Table 3:** Test of antagonistic activities of *Bacillus licheniformis* HSW-16 against bacterial and fungal pathogens.

Bacteria	Zone of inhibition (mm)
*Escherichia coli*	15.9 ± 0.35
*Staphylococcus aureus*	NZ
*Bacillus cereus*	NZ
*Erwinia carotovora*	16.6 ± 0.29
*Klebsiella pneumoniae*	14.3 ± 0.51
*Enterobacter* sp.	13.9 ± 0.43
**Fungal species**	
*Fusarium oxysporum*	17.1 ± 0.45
*Fusarium moniliforme*	NZ
*Fusarium graminearum*	16.8 ± 0.56
*Aspergillus flavus*	9.7 ± 0.14
*Candida albicans*	NZ
*Colletotrichum casici*	NZ
*Penicillium citrinum*	15.2 ± 0.38

*±Standard deviation; NZ, no zone of inhibition*

### Effect of HSW-16 on Plant Growth Under NaCl Stress

#### Physicochemical Characteristics of Soil and Plant Growth Studies

**FIGURE 4:**
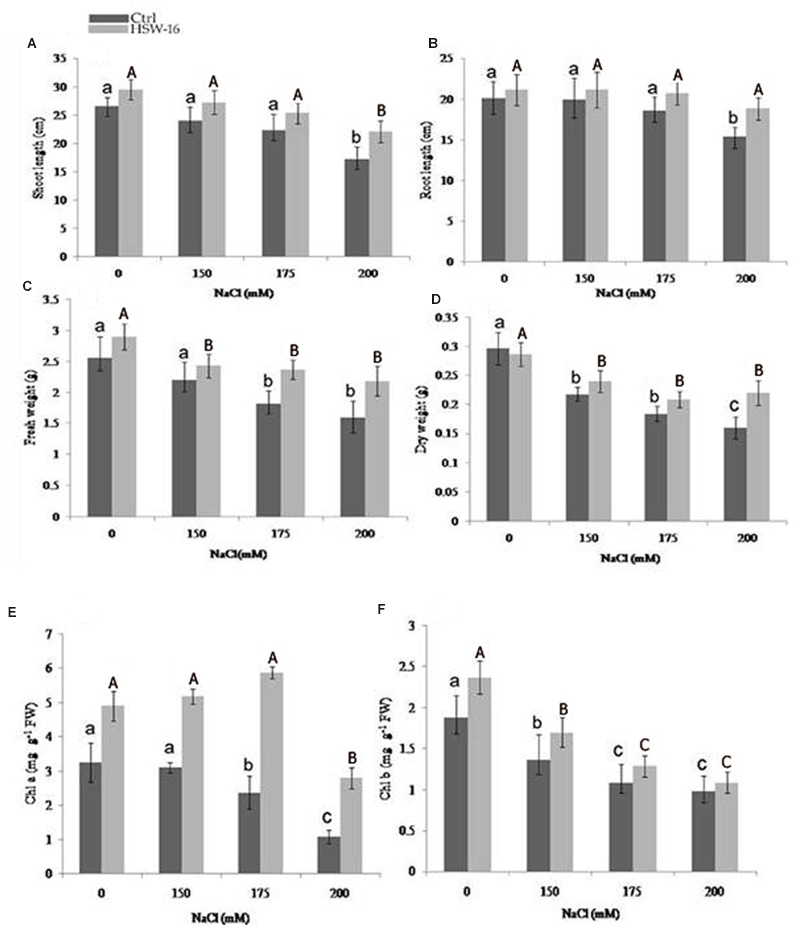
**Effect of inoculation with *B. licheniformis* HSW-16 on plant biomass and chlorophyll content under different salinity conditions (0 mM, 150 mM, 175 mM, 200 mM NaCl). (A)** Shoot length, **(B)** root length, **(C)** fresh weight, **(D)** dry weight, **(E)** chlorophyll a, and **(F)** chlorophyll b. Small letters on the bar in each treatment indicate significant difference (*p* < 0.05) among control, whereas capital letters indicate significant difference among the bacterial treatment. Dark and light gray columns represent the control (Ctrl) and HSW-16 inoculated plants, respectively.

The soil was analyzed for its nutritional status for the plant growth study. The various parameters of soil were as follows: pH 7.20, EC 0.162ds m^-1^, Olsen P 34.6 mg kg^-1^, OC (organic C) 0.2%, total N 61 mg kg^-1^, K 140.6 mg kg^-1^, Zn 0.218 mg kg^-1^, Cu 0.124 mg kg^-1^, Fe 2.61 mg kg^-1^, and Mn 0.948 mg kg^-1^. To evaluate the effect of salt stress in control and HSW-16-treated plants, we carried out Student’s *t*-test (*p* < 0.05), which demonstrated that the inoculation of HSW-16 resulted in significant increase in wheat plants growth under different concentration of NaCl. The growth was assessed by measuring shoot length, root length, fresh weight, and dry weight. In presence of bacterium, the shoot length increased significantly by 27.82% at 200 mM (1.17%) of NaCl as compared to uninoculated plants, whereas, it increased 13.26 and 13.35% at 150 and 175 mM of NaCl, respectively (**Figure 4A**). Similarly, a significant increase in root length (22.55%) was recorded at 200 mM of NaCl in response to bacterium inoculation. However, root length increased only by 11.13 and 6.18% at 175 mM and 150 mM of NaCl, respectively, in inoculated plants (**Figure 4B**). The results of fresh weight showed that HSW-16 increased fresh weight significantly at 200 and 175 mM of NaCl by 37.11 and 30.94%, respectively (**Figure 4C**). Data of dry weight of experimental plants also indicated that inoculation of HSW-16 significantly increased dry weight at 200 mM (37.5%) in comparison to their control plants treated with NaCl alone (**Figure 4D**). A remarkable increase in chlorophyll a content was recorded in HSW-16 inoculated plants which showed 159.26, 147.68, and 67.09% increase at 200, 175, and 150 mM of NaCl, respectively, over uninoculated plants. It also significantly increased the content of chlorophyll b by 26.06% than that of control plants treated with 0 mM of NaCl (**Figures 4E,F**).

#### Inoculation Effect on Na^+^, K^+^, and Ca^2+^ Ions Under NaCl Stress

**FIGURE 5:**
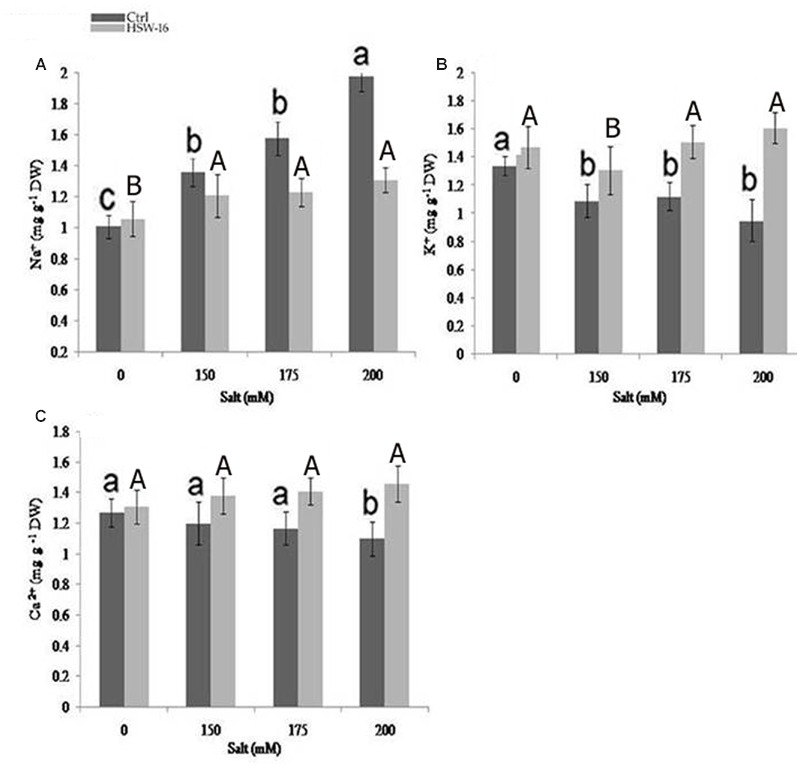
**Effect of NaCl and inoculation with *B. licheniformis* HSW-16 on ionic uptake by plants (A)** Na^+^ **(B)** K^+^ **(C)** Ca^+2^. Each value is mean of five replicates. Error bar represents standard error of five replicates of mean. Small letters on the bar in each treatment indicate significant difference (*p* < 0.05) among control, whereas capital letters indicate significant difference among the bacterial treatment. Dark and light gray columns represent the control (Ctrl) and HSW-16 inoculated plants, respectively.

To study the role of PGPB in ameliorating NaCl stress, plants were evaluated for fine-tuning of the ionic balance, Na^+^/K^+^ ratio in particular, using Atomic Absorption Spectrometry (AAS). There was a significant change in the ionic constituents of wheat seedlings grown under NaCl stress. Ionic analysis demonstrated a decrease in Na^+^ content but an increase in K^+^ and Ca^2+^ content in plants inoculated with test isolate HSW-16. In comparison to uninoculated control plants, inoculated plants exhibited the highest decrease in Na^+^ (51.14%) and increase in K^+^ content (68.42%) at 200 mM of NaCl. However, less change in Na^+^ and K^+^ were evident in plants grown at 150 and 175 mM (12.40%, 20.18%) of NaCl (**Figures 5A,B**). Moreover, the higher increase in Ca^2+^ (32.72%) was also observed at 200 mM of NaCl than the other concentrations, which was 20.51% and 15% at 175 and 150 mM of NaCl, respectively (**Figure 5C**).

#### Biochemical Analysis of Plants Treated with NaCl

**FIGURE 6:**
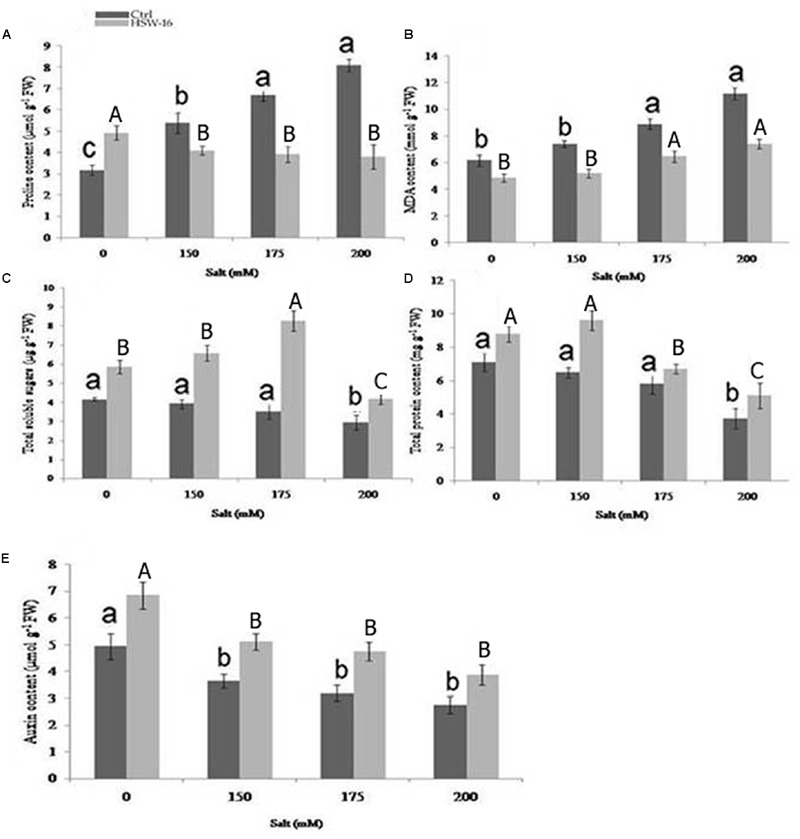
**Effect of *B. licheniformis* HSW-16 inoculation on (A)** proline **(B)** malondialdehyde (MDA) **(C)** total soluble sugar (TSS) **(D)** total protein, and **(E)** auxin content under different 0 mM, 150 mM, 175 mM, 200 mM NaCl salinity conditions. Values are mean of five replicates ± SD. Small letters on the bar in each treatment indicate significant difference (*p* < 0.05) among control, whereas capital letters indicate significant difference among the bacterial treatment. Dark and light gray columns represent the control and HSW-16 inoculated plants, respectively.

The change in certain chemical components namely, proline, MDA content, TSS, IAA, and TPC known to play important protective roles during NaCl stress was assessed in wheat plants with or without bacterial inoculation. Differential levels of above components were detected in plants inoculated with or without bacterium under NaCl stress. As the NaCl concentration increased, proline content increased in the range of 36.90 to 93.70% in uninoculated plants. However, the content of proline decreased in bacterium inoculated plants subjected to NaCl stress (**Figure 6A**). Significant decrease in proline content (53%) was observed at 200 mM of NaCl, followed by 175 mM (41.50%) and 150 mM of NaCl, as compared to un-inoculated control (**Figure 6A**). Since, NaCl-induced oxidative damage to lipids is reflected by increased MDA content, the level of MDA was estimated in plants. The amount of MDA content increased with increase in NaCl concentration in untreated plants. The increase in MDA content was 80.87% at 200 mM of NaCl as compared to the control plants. However, its concentration decreased under NaCl in plants that were inoculated with HSW-16. The maximum decrease in MDA content (50%) was observed at 200 mM of NaCl as compared to plants treated with same concentration of NaCl. Furthermore, decrease in MDA content was also observed at 150 mM (42.30%) and 175 mM of NaCl (37.56%) in bacterium inoculated plants as compared to control plants (**Figure 6B**)

A notable difference was found for TSS in wheat leaves stressed with different NaCl concentrations. The highest accumulation of TSS (67.51%) was observed at 150 mM of NaCl in HSW-16 inoculated plants as compared to control subjected to the same stress condition. The increase in TSS in HSW-16 inoculated plants was 52.54% and 41.90% at 175 and 200 mM of NaCl as compared to their control. However, salinity significantly reduced the TSS content in uninoculated plants. The highest decrease was 41.21% in control plants under 200 mM of NaCl as compared to control (**Figure 6C**). Similarly, inoculation of bacterium significantly improved TPC in plants under NaCl. The highest increase in protein content (48.15%) was observed at 150 mM of NaCl as compared to other concentrations (**Figure 6D**). HSW-16 inoculation significantly increased the protein content (38.46%) as compared to control plants under non-saline stress. Inoculation with test isolate HSW-16 also improved auxin content in wheat leaves under NaCl. The significant increase in auxin content was in the range of 38 to 49% in bacterium-inoculated plants under different concentration of NaCl. The highest increase in auxin content (48.90%) was at 175 mM of NaCl in inoculated plants as compared to respective control (**Figure 6E**).

#### EPS Quantification

The test organism was able to produce 3.09 ± 0.07 μg ml^-1^ EPS in normal growth medium. IR spectroscopy of EPS extracts showed characteristic absorption bands indicating the presence of hydrogen-bonded compounds. Characteristic β-1,3-glucan band in crude exopolysaccharide was observed between 1000-1500cm^-1^ (1100, 1200, 1400, and 1600 cm^-1^), whereas alkane was observed at 2929 cm^-1^ (**Supplementary Figure S2**).

#### Confirmation of Colonization

The efficiency of colonization by HSW-16 was determined by plate counting, microscopic evaluation, and ERIC-PCR of inoculated bacteria with respect to control after 15 days of growth. After the experimental period, associated bacterium was found in a range of 1.2 × 10^3^ CFU g^-1^ of the root. Colonization of bacterium was also tested by acridine orange staining, which showed a large number of fluorescent cells on the surface of plant root (**Supplementary Figure S3A**). No bacterial cells were observed on the root surface of uninoculated plants. Additionally, to confirm the identity of colonized bacterium, total DNA of inoculated plants was subjected to ERIC-PCR. ERIC-PCR profile obtained from total DNA of inoculated plants was identical to that of a pure culture of test isolate, which indicated that bacterium colonized plants successfully (**Supplementary Figure S3B**).

## Discussion

Some plant growth promoting bacteria can help plants combat salt stress through various means. One of the important mechanisms for ameliorating salt stress in plants is mediated by production of ACC deaminase (ACCD), which causes a decrease in the level of ‘stress ethylene’ produced in plants in response to stress (Glick, 2007; Saleem et al., 2007). The present study demonstrates the effectiveness of ACC deaminase bacterium *B. licheniformis* HSW-16 for improving growth of wheat plants under salt stress conditions. Inoculation of this bacterium also resulted in balancing Na^+^/K^+^ ratio in plants and the production of osmolytes, which protect plants from harmful effects of salinity. Thus, our report extends the understanding of plant growth promoting properties contributed by members of genus *Bacillus*. The plant growth promoting effect of *Bacillus* sp. on wheat plants under different salinity conditions has also been observed previously by Upadhyay et al. (2012). In the present study, *B. licheniformis* HSW-16 was recovered from the salt-enriched water of Sambhar salt lake located in Rajasthan, India. Isolation of salt tolerant ACC deaminase bacteria from above location has been reported in earlier studies. Based on the qualitative screening, Sahay et al. (2012) reported the isolation of a few bacterial sp. including *Bacillus* sp., *B. licheniformis, B. marisflavi, B. baekryungensis, B. selenatarsenatis, Thalassobacillus devorans, Halomonas salina, Oceanobacillus picturae*, and *Streptomyces radiopugnans* from Sambhar salt lake which were ACCD positive.

Selection of test isolate was primarily based on high ACCD activity as well as other plant growth promoting properties. The level of ACCD activity considered for selection was based on the earlier studies which suggest that the bacteria producing >20 nmol α-KB mg^-1^ protein h^-1^ can enhance plant growth by reducing the level of stress ethylene produced in plants under stress conditions (Penrose and Glick, 2003). This was corroborated by results of *Arthrobacter protophormiae* having ACC deaminase activity of 241 nmol α-KB mg^-1^protein h^-1^, which alleviated the damages caused by NaCl stress, and resulted in higher yield (Barnawal et al., 2014).

Furthermore, the presence of ACCD in test organism was confirmed by amplification of *AcdS*, a gene encoding ACC deaminase in bacteria. The universal primers used in this study have also been employed for successful amplification of *AcdS* gene in different environmental isolates (Jha et al., 2012; Shrivastava and Kumar, 2013). The nucleotide sequence of 800 bp amplicons was confirmed by sequence analysis, which matched with the *AcdS* of other bacteria available in the database. Phylogenetic analysis suggested that the *AcdS* gene sequence of HSW-16 was similar to bacteria belonging to genus *Bacillus* as well as other bacterial genera. To the best of our knowledge, this is the first report where the presence of *AcdS* in *B. licheniformis* has been demonstrated. Phylogenetic analysis indicated similarity of *AcdS* sequences among diverse species including *Pseudomonas* sp., *Klebsiella* sp., and *Serratia* sp. This observation is supported by a recent report which illustrates that the evolution of ACCD occurred mainly through vertical transfer with occasional horizontal transfer (Nascimento et al., 2014).

In addition to ACCD activity, test isolate HSW-16 also showed other plant growth promoting properties such as the production of phytohormone (auxin) and phosphate solubilization activity which can benefit plant growth and productivity. The ability of ACCD bacteria equipped with such features to benefit host plants has been demonstrated in several studies (Sachdev et al., 2009; Karthikeyan et al., 2012). Inoculation of IAA-producing bacteria promotes root growth by increasing number and length of adventitious root as well as by altering root architecture that lead to enhanced nutrient uptake (Chakraborty et al., 2006; Yang et al., 2009; Dutta et al., 2015), which in turn promotes plant growth (Chakraborty et al., 2006; Yang et al., 2009; Dutta et al., 2015). Similarly, phosphate solubilization is an important attribute of rhizospheric microorganism that provides the bio-available phosphate for improvement of plant growth (Liu et al., 2014; Dutta et al., 2015). The presence of phosphate solubilizing bacterium in soils may be considered a positive indicator of utilizing the microbes as biofertilizers for crop production and beneficial for sustainable agriculture. The nitrogen fixation ability of associative bacteria provides the essential nitrogen content during the growth phase of the plant. The nitrogen fixation ability is evident from the amplification of *nifH* gene, that has been demonstrated in several associative as well as endophytic bacteria by using universal primers (Desnoues et al., 2003; Jha and Kumar, 2009)

Bacterial inoculation under laboratory conditions significantly enhanced plant growth with respect to different parameters tested. A significant decrease in shoot/root lengths and fresh/dry weight was observed in uninoculated plants under salt stress, whereas inoculation with HSW-16 limited these losses significantly. It is likely that this response might be due to ACCD activity of the bacterium. Our results are in agreement with the previous report for salt tolerance in various plants induced by PGPR (Mayak et al., 2004; Saravanakumar and Samiyappan, 2007; Zahir et al., 2009). Effect of salinity stress is manifested by a decrease in K^+^ availability and its reduced transportation to growing region of plants (Grattan and Grieve, 1999). As the Na^+^ ion level increases, ionic competition results in increased Na^+^/K^+^ ratio, thus having significant negative effect on plant growth (Schachtman and Liu, 1999). Moreover, the ability of Na^+^ to bind competitively with K^+^ binding site disrupts the normal cellular function and generates metabolic toxicity (Bhandal and Malik, 1988; Ruiz-Lozano and Azcón, 2000). The altered ionic ratio of Na^+^ and K^+^ generated on accumulation of NaCl in plants can be improved by certain associative bacteria. Some PGPR help plants indirectly by strengthening their ability to combat salt stress (Nadeem et al., 2006). Therefore, plants growing under salt stress were also tested to see whether there is change in plants ability to prevent or minimize accumulation of Na^+^ in plant tissue on inoculation of HSW-16. The result of ion analysis showed that the test organism decreased Na^+^ accumulation and increase in K^+^ content in plants. These results are in agreement with by earlier studies which suggest that certain PGPR not only delay the uptake of Na^+^ but also promote the extrusion of excess Na^+^ from the shoot (Arshad et al., 2009). These PGPR up-regulate the expression of HKT1 (High affinity K^+^ transporter 1) gene which helps in Na^+^ eﬄux in the plant. The differential regulation of HKT1 expression in root and shoot results in reduced accumulation of Na^+^, and an increased accumulation of K^+^ under salt stress conditions (Zhang et al., 2008). Similarly, PGPB *B. subtilis* GB03 induced concomitant down- and up-regulation of HKT1 expression in roots and shoots of *Arabidopsis* seedlings, respectively (Zhang et al., 2008). Moreover, secretion of exopolysaccharides by HSW-16 also could be important for reducing Na^+^ uptake in the plant by sequestering it as suggested by earlier reports (Roberson and Firestone, 1992; Qurashi and Sabri, 2012).

Accumulation of compatible solutes (osmolytes) in response to bacterial inoculation helps to stabilize the osmotic adjustment (Ashraf and Harris, 2004). These compatible solutes are highly soluble and are usually nontoxic at high cellular concentrations (Hasegawa et al., 2000; Ashraf and Foolad, 2007). The level of compatible solutes or osmolytes significantly increased in response to salinity stress (Hasegawa et al., 2000; Chen and Murata, 2008). Increase in proline content helps to stabilize the membranes and proteins, buffers the redox potential, and acts as hydroxyl radical scavenger under salt stress (Claussen, 2005). Furthermore, among the various compatible solutes, proline accumulation is the most frequently reported modification induced by salt stress in plants (Ashraf and Foolad, 2007). In the present study, proline levels were considerably higher in uninoculated plants subjected to varying level of salt stress. However, bacterial inoculation significantly decreased the proline content indicating a lower degree of stress severity in plants under salt stress. This suggests that PGPR-inoculated plants do not face much NaCl stress, therefore, the proline accumulation is less in HSW-16 inoculated plants. In addition to proline, soluble sugars also remarkably maintain the osmotic homeostasis and constitute about 50% of the total osmotic potential in plant cells during NaCl stress (Cram, 1976). The increase in TSS content in inoculated plants illustrates the survival of plants under saline conditions. The protein content was also higher in inoculated plants growing with or without NaCl that might have correlation with salinity tolerance. Previous report of Kandasamy et al. (2009) has shown the differential expression of protein in rice plants after inoculation with *Pseudomonas fluorescens*.

Malondialdehyde content reflects the extent of stress as well as oxidative damage caused by ROS generated by NaCl (Jain et al., 2001). Under NaCl stress, enhanced production of MDA as a consequence of decomposition of polyunsaturated fatty acids of bio-membranes has been reported (Gossett et al., 1994). In the present study, we observed that inoculation with test isolate caused a reduction in MDA content in inoculated plants as compared to uninoculated plants. Decrease in MDA content following inoculation of test organism points to cause lesser cell membrane damage and/or induce enhanced salinity tolerance to plants.

Maximum benefit from rhizobacterial inoculation depends on efficient colonization of bacteria in plants. Therefore, the efficiency of colonization of bacterium was tested employing multiphasic approaches. Photomicrograph of roots of plants inoculated with the bacterium and stained by acridine orange showed large number of bacterial cells adhering to the roots. It suggested that this isolate can effectively colonize plant surface (Hobbie et al., 1977; Olsen and Bakken, 1987; Morris et al., 1997). Root colonization is the first and foremost step for plant-microbe association in which microorganisms move towards rhizosphere in response to root exudates. Thus, motility and chemotaxis play a key role in the root colonization. The test isolate HSW-16 showed all forms of motility which is required for chemotactic responses and colonization on plant surface (Van de Broek et al., 1998; Lugtenberg et al., 2001). The role of motility by PGPR *Pseudomonas fluorescens* and *Bacillus* sp. has been demonstrated in previous studies (Misaghi et al., 1992; Bashan and Holguin, 1995; Lugtenberg and Kamilova, 2009). Moreover, recovery of bacterial colonies from inoculated plants showed that HSW-16 was efficient to colonize plants (Anith, 2009; Joshi and Bhatt, 2011). Additionally, results of ERIC-PCR confirmed the identity of recovered colonies as HSW-16 and provided evidence for successful colonization of the roots of the plant. Ability of this isolate to colonize and benefit other plants needs to be tested in detail.

## Conclusion

The present work aimed to study the role of halotolerant ACC deaminase bacterium *B. licheniformis* HSW-16 to evaluate the plant growth under salt stress. Besides ACCD activity, the test isolate possesses other plant growth promoting properties including IAA production, and, phosphate solubilization that could mitigate salt stress-induced damages and establish ‘induced systemic tolerance’ to the plants. Inoculation of test isolate HSW-16 improved growth of the wheat plants growing under NaCl. In addition, inoculation with bacterium also improved the compatible solutes to counteract the NaCl stress. Our results provide evidence of colonization ability of HSW-16 that may be required for improvement of plant growth and regulation of ion transporters favoring amenable K^+^/Na^+^ ratio. In conclusion, this work opens up the possibility to evaluate the potential of *B. licheniformis* HSW-16 under field conditions as biofertilizer in alleviating salinity stress faced by the plants.

## Author Contributions

RPS performed the experiments and wrote a draft of the manuscript. PNJ mentored the research work and revised the manuscript.

## Conflict of Interest Statement

The authors declare that the research was conducted in the absence of any commercial or financial relationships that could be construed as a potential conflict of interest.

## Footnotes

1 http://www.ncbi.nlm.nih.gov/BLAST

2 http://www.ncbi.nlm.nih.gov

## Supplementary Material

FIGURE S1 **Amplification of *nifH* gene in *Bacillus licheniformis* HSW-16**.

FIGURE S2 **Characterization of the EPS components of *B. licheniformis* HSW-16 by Fourier transform-infrared spectrograms (FTIR-spectroscopy)**.

FIGURE S3 **Efficiency of colonization of *B. licheniformis* HSW-16 was assessed 15 days after germination of bacterized wheat plants (A)** visualization of acridine orange-stained wheat plant roots showing bacterial cells using fluorescence microscopy **(B)** ERIC-PCR profile of bacterium colonized in wheat plants and confirmation of its identity using pure culture (Lane M: DNA ladder SM0311, Lane C: control DNA, Lane HSW-16: DNA of *B. licheniformis* isolated from inoculated plant).
