# Supplementary figures and images for: A Halotolerant Bacterium Bacillus licheniformis HSW-16 Augments Induced Systemic Tolerance to Salt Stress in Wheat Plant (Triticum aestivum)
Source: Front Plant Sci. 2016 Dec 16;7:1890. doi: 10.3389/fpls.2016.01890 (PMC5159429; doi:10.3389/fpls.2016.01890)

**Suppl. Fig. 1**

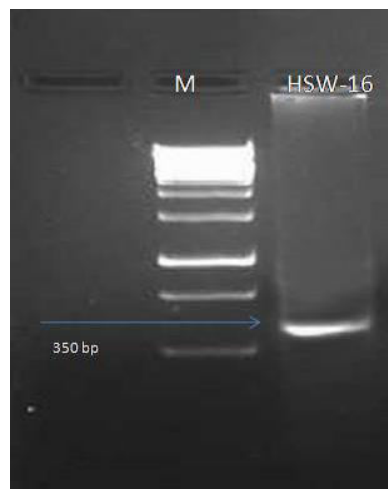

**Suppl. Fig.2**

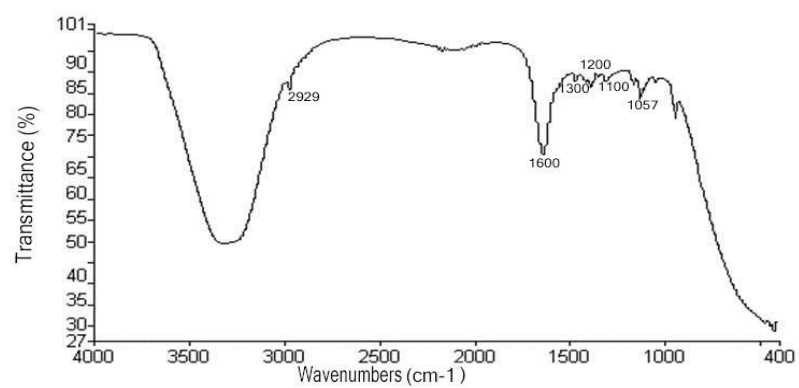

**Suppl. Fig. 3**

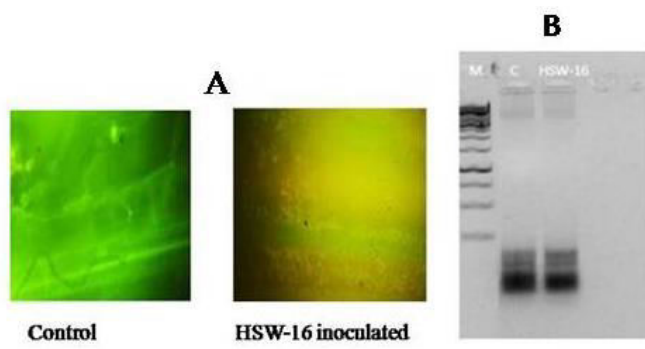

Supplement: FIGURE S1 — Amplification of nifH gene in Bacillus licheniformis HSW-16. [file Data_Sheet_1.PDF]
